# ATM/CHK/p53 Pathway Dependent Chemopreventive and Therapeutic Activity on Lung Cancer by Pterostilbene

**DOI:** 10.1371/journal.pone.0162335

**Published:** 2016-09-09

**Authors:** Hani Lee, Yonghwan Kim, Ji Hye Jeong, Jae-Ha Ryu, Woo-Young Kim

**Affiliations:** 1 Research Center for Cell Fate Control, College of Pharmacy, Sookmyung Women’s University, Seoul, 04310, Republic of Korea; 2 Department of Biological Sciences, Sookmyung Women’s University, Seoul, 04310, Republic of Korea; German Cancer Research Center, GERMANY

## Abstract

Among the many stilbenoids found in a variety of berries, resveratrol and pterostilbene are of particular interest given their potential for use in cancer therapeutics and prevention. We purified four stilbenoids from *R*. *undulatum* and found that pterostilbene inhibits cancer cell proliferation more efficiently than rhapontigenin, piceatannol and resveratrol. To investigate the underlying mechanism of this superior action of pterostilbene on cancer cells, we utilized a reverse-phase protein array followed by bioinformatic analysis and found that the ATM/CHK pathway is modified by pterostilbene in a lung cancer cell line. Given that ATM/CHK signaling requires p53 for its biological effects, we hypothesized that p53 is required for the anticancer effect of pterostilbene. To test this hypothesis, we used two molecularly defined precancerous human bronchial epithelial cell lines, HBECR and HBECR/p53i, with normal p53 and suppressed p53 expression, respectively, to represent premalignant states of squamous lung carcinogenesis. Pterostilbene inhibited the cell cycle more efficiently in HBECR cells compared to HBECR/p53i cells, suggesting that the presence of p53 is required for the action of pterostilbene. Pterostilbene also activated ATM and CHK1/2, which are upstream of p53, in both cell lines, though pterostilbene-induced senescence was dependent on the presence of p53. Finally, pterostilbene more effectively inhibited p53-dependent cell proliferation compared to the other three stilbenoids. These results strongly support the potential chemopreventive effect of pterostilbene on p53-positive cells during early carcinogenesis.

## Introduction

Despite advances in our understanding of the molecular mechanisms of carcinogenesis, cancer remains one of the leading causes of death worldwide.[1] Accordingly, considerable attention has been focused on strategies of cancer prevention. One of such is chemoprevention, which involves preventing carcinogenesis or delaying of cancer progression through taking of dietary or pharmaceutical agents.[2–6] Carcinogenesis is a multistep process that involves accumulation of genetic alterations accompanying the progression of pre-malignant lesions to malignancy.[7–9]

As chemical compounds that occur naturally in plants, phytochemicals exhibit potent anti-mutagenic and anti-carcinogenic properties.[10–12] To date, investigations of the chemopreventive effects of phytochemicals have been primarily focused on their antioxidant activities in reducing oxidative stress and thus decreasing cellular DNA damage.[13, 14] Another possible chemopreventive strategy involves preventing the precancerous to cancer transition via activation of p53-dependent senescence or apoptosis in precancerous cells; however, this possibility has thus far not been intensively investigated.[15–17]

Pterostilbene (*trans*-3,5-dimethoxy-4’-hydroxystilbene; PT) is a naturally derived phytoalexin.[18] This dimethylated analogue of resveratrol is found in blueberries, some grapes and tree wood. Due to their structural similarities, PT exhibits pharmacological functions that are similar to well-studied resveratrol, including anti-oxidant, anti-inflammation, anti-cancer and anti-diabetes activities.[19] Resveratrol contains three hydroxyl groups, whereas PT has two methoxy groups and one hydroxyl group; the two methoxy groups are responsible for the relatively increased lipophilicity and enhanced cell membrane permeability of PT. Furthermore, pharmacokinetic analyses have revealed that PT has a longer half-life than resveratrol when administered orally.[18] Indeed, the *in vitro* pharmacological activities of PT are more potent than those of resveratrol in various settings.[20] The anti-tumor activities of PT are mediated by multiple molecular targets based on cancer cell type and are characterized by cell cycle arrest or cell death. However, these cellular responses may result from genomic instability upon treatment with PT, and it remains unclear whether PT acts as a genotoxic agent. Treatment of cancer cells with resveratrol or PT induces cell cycle arrest and DNA damage, indicating that both phytochemicals act as genotoxic agents.[21–24] Recently, it was reported that resveratrol might function as a topoisomerase II poison, suggesting that resveratrol could generate stalled replication forks during S phase.[25–27] However, whether the anti-cancer activity of PT involves induction of replication stress remains unknown.

Faithful DNA replication is crucial for the inheritance of genetic information as well as for maintaining genome integrity. Experimental evidence indicates that a sizable amount of spontaneous DNA damage occurs during S phase,[28] and when faced with numerous lesions, the replication machinery stalls and replication forks collapse, leading to DNA damage. Failure to repair replication-associated DNA damage activates multifaceted DNA damage responses, which result in cell cycle arrest, cellular senescence or cell death.[29] The kinases Ataxia Telangiectasia and Rad3-related protein (ATR) / Ataxia telangiectasia mutated (ATM) and Checkpoint Kinase 1/2 (CHK1/2) constitute the critical DNA damage response module at stalled replication forks, which is characterized as replication stress.[30] Activated ATM/ATR phosphorylates CHK1/2, resulting in the activation of downstream effector molecules, including p53, followed by full activation of the replication stress response. Therefore, due to the continuous proliferative pressures of precancerous and cancer cells, the cellular response to replication stress could serve as a potent chemotherapeutic target.[31, 32] Various chemotherapeutic agents, including hydroxyurea and topoisomerase poisons, lead to stalled replication forks via different mechanisms of action.[33]

In this study, we investigated the therapeutic and preventive effect of PT on non-small cell lung cancer cell (NSCLC, A549) and precancerous human bronchial epithelial cell (HBEC) lines using stilbenoids purified from the roots of *R*. *undulatum*, which has long been used in traditional medicine in Asia. Here, we demonstrate that PT activates the ATM/CHK/p53-dependent pathway, leading to p53-dependent senescence in lung premalignant cells. The results suggest that PT might mediate its chemopreventive action in cells in which p53 is still active during the early stage of lung carcinogenesis.

## Materials and Methods

### Stilbenoids purification

Stilbenoids were purified from *R*. *undulatum* as described previously.[34] Briefly, the methanolic extract of the dried rhizoma of *R*. *undulatum* was suspended in water and partitioned with ethyl acetate. The ethyl acetate soluble fraction was subjected to column chromatography on silica gel eluting with n-hexane-ethyl acetate (20:1→1:1) and CHCl_3_-MeOH(10:1→1:1) to obtain six fractions. From these fractions, pterostilbene, rhapontigenin, resveratrol, and piceatannol were isolated with column chromatography and the structure of isolated compounds were conformed as described previously. [34] The pterostilbene purchased from Sigma Aldrich (Saint Louis, MO) were also used.

### Cell lines and chemicals

Human A549 lung adenocarcinoma cells were purchase from ATCC (Manassas, VA) and cultured in RPMI-1640 medium (Gibco, Grand Island, NY) supplemented with 10% fetal bovine serum (with 25mM HEPES, Gibco, Grand Island, NY) and penicillin and streptomycin (100unit/ml, Gibco, Grand Island, NY). Two immortalized human bronchial epithelial cell lines (HBECs) were used.[35] Both of cell lines express oncogenic form of Kras^V12^. HBECR express intact p53 while HBECR/p53i suppressed p53 expression by shRNA against p53 described previously and gifted by Dr. Minna.[36, 37] These cells were cultured as described.[35] Cells were maintained at 37°C in a 5% CO_2_/95% air incubator. All chemicals not mentioned separately were purchased from Sigma Aldrich (Saint Louis, MO).

### Analysis of cell viability

A cell counting assay was performed for analysis of cell viability. A549 (1.5×10^3^) and HBECR or HBECR/p53i (2×10^3^) Cells were seeded into 96well plates and cultured overnight. Cells were treated with pterostilbene by exchanging medium premixed with different concentration for 72hr. After cells were stained with Hoechest 33258 (5ng/ml, Invitrogen, Grand Island, NY) for 10 minutes at room temperature in the dark, cell were counted by an image reader Cytation3 (Biotek, Winooski, VT). Results are expressed as percent relative to vehicle treated cells.

### Cell cycle analysis

A549 (3×10^5^) and HBECRs (5×10^5^) cells were seeded into 60mm dishes and cultured overnight. To examine the cell cycle, well growing A549 cells cultured in the medium containing 10% serum or no serum were treated with indicated concentrations of pterstilbene and followed by further cultures for 48hrs. To explore the effect of pterostilbene on the double thymidine block cell cycle transition, HBECRs cells were synchronized by thymidine at G1-early S phase. To synchronize at G1-early S phase, cells were treated with 2mM thymidine first block for 3hr, released thymidine-free medium for 3hr, treated with 2mM thymidine second block for 18hr and released with vehicle or pterostilbene. Then cells were harvested at the indicated time point, fixed with 70% ethanol, and incubated with propidium iodide (25μg/ml, Sigma Aldrich, Saint Louis, MO) for 10 minutes. The cell cycle distribution was measured by (FACS Calibur, BD, San Jose, CA) and cell cycle profiles were obtained with ModFit LT software (Verity Software House, Topsham, ME). The cell cycles were further examined with labeling of S-Phase with BrdU followed with detection with BrdU-FITC Antibody (BD, San Jose, CA) and flow cytometry, FACS Calibur. Cell proliferation assay was performed with ELISA BrdU kit (11647229001, Roche) according to the owner’s instruction.

### Reverse Phase protein Array (RPPA)

A549 (3×10^5^) cells were seeded into 6 well plates and cultured overnight. Cells were starved in serum free medium for 48hr and treated with 2.5μM, 5μM of pterostilbene for 72hr and all samples were quadruplicated. Total lysates were prepared with protocol of Proteomic Core of MD Anderson Cancer Center and the RPPA assay was performed in. the same center. The data were analyzed by using the Cluster and TreeView programs to generate Hierarchical clustering and the heat map.

### Western Blotting and immunofluorescence staining

The cells were lysed with modified RIPA buffer containing 50mM Tris-HCl pH 7.4, 1% NP40, 1% triton X-100, 0.25% sodium deoxycholate, 150mM sodium chloride, 10% glycerol,protease inhibitors (complete mini, Roche, South San Francisco, CA), and phosphatase inhibitors (1mM Na_3_VO_4_, 1mM NaF, 20mM β-glycerophosphate) and sonicated. The cleared lysates by centrifugation was resolved in SDS-polyacrylamide gel by electrophoresis and then transferred to a PVDF membrane (Bio-rad, Hercules, CA). The membranes were blocked by 5% non-fat dry milk in a buffer containing 20mM Tris pH7.4, 150mM NaCl and 0.1% Tween-20, then probed with primary antibodies against phospho-ATM, phospho-chk1, phospho-chk2, phospho-p53, p21 (all were obtained from Cell Signaling Technology, Danvers, MA) and p53 (Santa Cruz Biotechnology, Santa Cruz, CA). GAPDH was used as loading control. Western blots were visualized with chemiluminescence-based immunodetection of horse radish peroxidase and detected Supersignal West Femto (Thermo, Waltham, MA), Amersham (GE healthcare, Piscataway, NJ), EzWest Lumi (Atto corporation, Tokyo, Japan), or Novex (Invitrogen, Grand Island, NY). Antibodies to phospho-H2AX (γH2AX) and p53BP were purchased from Cell Signaling Technology (Danvers, MA), and Millipore (Billerica, MA) respectively. The cells were cultured on 4 well chamber slides (Thermo Scientific, Waltham, MA) and drugs were treated after 24hrs. The cells were fixed with cold methanol and then treated with blocking solution (5% BSA in buffered saline) and the primary antibodies and fluorescence labeled secondary antibodies were treated. The images were taken under Leica TCS SP8 STED 3X super-resolution microscope (Wetzlar, Germany)

### Senescence measurements.μ

The induction of cell senescence in HBEC cells by PT was measured as described previously.[38, 39] The drugs were treated for 72 hrs to the cells, The senescence associated β-galactosidase positive cells were counted by microscopy after stained with 5-bromo-4-chloro-3-indolylP3-D-galactoside[39] or by FACS Calibur after stained with 5-dodecanoylaminofluorescein di-beta-D-galactopyranoside (C12FDG)[38].

## Results

### Effects of pterostilbene on cell proliferation and the cell cycle

**Fig 1:**
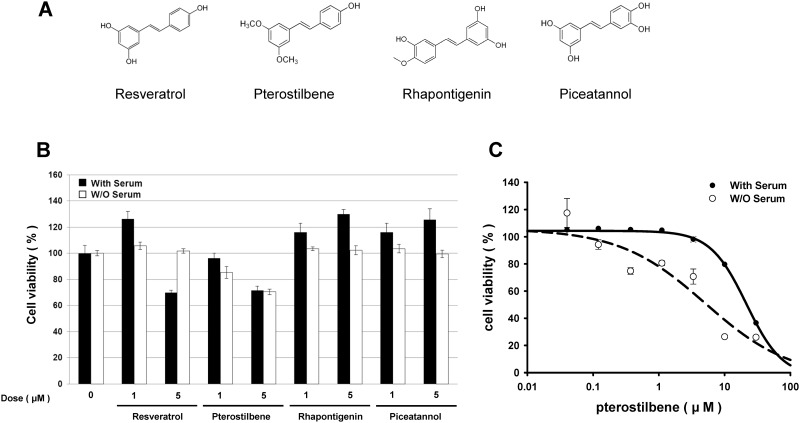
Effect of pterostilbene on cancer cell proliferation. (A) Chemical structures of 4 stilbenoids used. (B) A549 cells were seeded in 96-well cell plate. The cells were treated with resveratrol, pterostilbene, rhapontigenin and piceatannol at the final concentration of 1 μM and 5 μM in the presence and absence of FBS. After three days, the cell viability was determined by total live cell counting. (C) A549 cells seeded in 96 well plates were treated with different concentrations of pterostilbene ranging from 0 to 30 μM. After three days, the cell viability was determined by total live cell counting. *, *P < 0*.05; **, *P< 0*.*01* by Student’s *t* test.

Cancer-preventing effects of stilbenoids *in vitro* have been noted at relatively high concentrations, e.g., 50–100 μM.[18, 40, 41] However, as humans can only obtain a small amount of stilbenoids from dietary sources, including berries and grapes, the beneficial effects should be re-assessed at low stilbenoid concentrations. To this end, we treated the NSCLC line A549 with relatively low concentrations (1 and 5 μM) of four stilbenoids and examined the cytotoxic effects. The four stilbenoids were resveratrol, pterostilbene (PT), rhapontigenin, and piceatannol, which are very similar in structure (Fig 1A).

Treatment with low PT concentrations caused minimal inhibition of A549 cell growth, as previously demonstrated by others (data not shown). However, we did observe significantly enhanced cytotoxicity of A549 cells under conditions of serum withdrawal at low PT concentrations (1 and 5 μM) (Fig 1B). Conversely, the other stilbenoids did not suppress cell growth at these concentrations. We then examined the cytotoxic effect of various concentrations of PT in the presence and absence of serum and calculated IC50 values of approximately 21 μM and 5 μM, respectively (Fig 1C). This finding suggests that PT inhibits cell growth or survival in the absence of the various exocrine cell growth signaling factors included in FBS.

**Fig 2:**
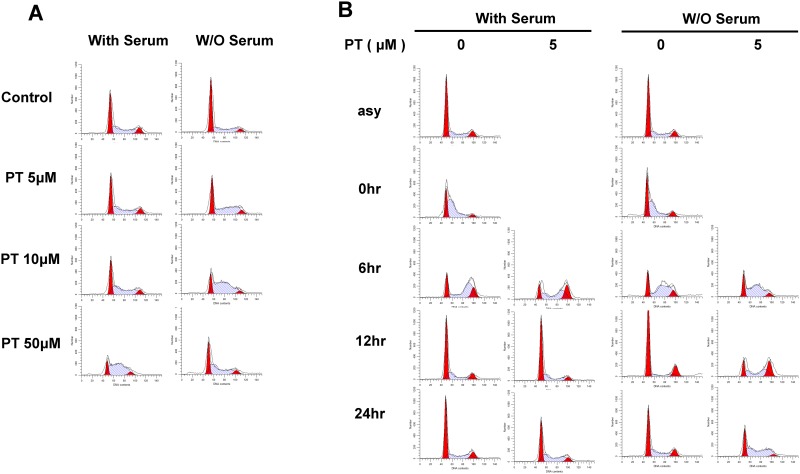
Effect of pterostilbene treatment on the cell cycle profile. (A) A549 cells in 60mm dishes cultured with or without FBS were treated with indicated concentrations of pterostilbene for 48hr and the cell cycle was analyzed. (B) For synchronization at G0 phase, A549 cells were treated 2mM thymidine for 18hr and released media without thymidine for 6hr. For second block, the cell were treated 2mM thymidine for 18hours and released indicated time with or without pterostilbene and the cell cycle was measured. The red areas represents G0/G1 or G2/M stage cells and the blue shaded area represents S-stage cells after analyzed by Modfit LT program from Verity Software (Topsham, ME).

To further understand the cytotoxic effects of PT, we analyzed the A549 cell cycle profile after PT treatment. Although serum deprivation slightly reduced the populations of S and G2/M cells, the cells continued to grow (Fig 2A), and cell synchronization revealed that serum deprivation slowed cell cycle progression (Fig 2B).

Consistent with the mild inhibition of cell growth (Fig 1C), treatment of A549 cells with relatively low concentrations of PT (5 and 10 μM) in the presence of 10% serum did not perturb cell cycle progression at 24 hr (Fig 2A). However, treatment of A549 cells with the same concentration of PT in the absence of serum induced S-phase arrest (Fig 2A, 5 and 10 μM). As reported previously, treatment of FBS-treated cells with a high concentration of PT (50 μM) disrupted cell cycle progression in a manner similar to the profile of cells treated with 10 μM of PT in serum-free medium.[42] In addition, synchronized cells exhibited a clearer difference with regard to the effect of PT treatment according to the presence or absence of serum. The length of the cell cycle was approximately 12 hr for cells exposed to serum, whereas it progressed slower for cells cultured without serum. Treatment with 5 μM PT only delayed the cell cycle under serum-free conditions (Fig 2B, 12 and 24 hr). These findings suggest that the cytotoxic effects of PT are potentially more effective under the condition without multiple growth factors from serum of bovine fetus, and that this effect may be attributed to S-phase cell cycle arrest.

### Proteomic analysis reveals activation of the ATR-CHK1/2 signaling pathway upon PT treatment

**Fig 3:**
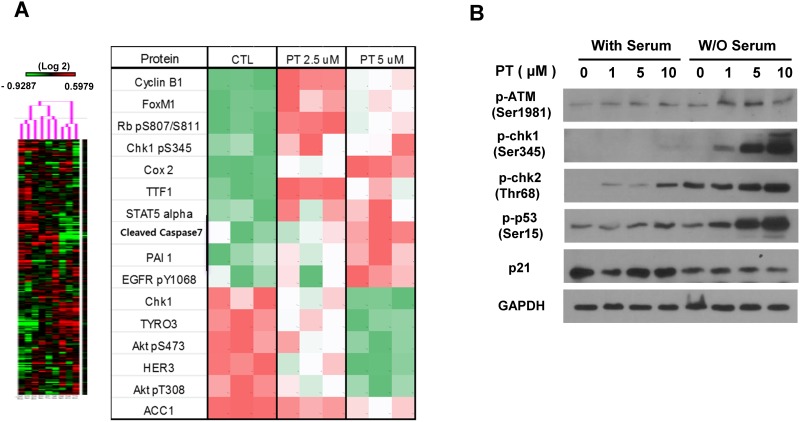
Pterostilbene activated ATR/CHK1/p53 pathway to arrests the cell cycle in A549. (A) Hierarchical clustering of RPPA data from A549 cells treated with pterostilbene at the concentration of 2.5 μM and 5 μM for 48 hours in serum free media (n = 4 for each samples). Protein expression levels of A549 without pterostilbene treatment were used as a control. The data are presented in a matrix format: rows represent individual protein features (186 proteins), and columns represent individual samples. Each cell in the matrix represents the expression level of a protein feature in an individual cell sample (left). A total of 16 proteins showing significant differences with *P* < 0.05 by Student’s *t* test are shown in the right panel. (B) Western blotting results of A549 cells treated with for the molecules implicated in DNA damage responses and cell cycle regulations.

We next employed reverse phase protein array (RPPA) analysis to investigate the underlying reasons for A549 cell growth inhibition and S-phase arrest upon treatment with relatively low concentrations of PT. As we observed enhanced cytotoxic effects and S-phase arrest under serum-depleted conditions, A549 cells cultured in serum-free medium were treated with DMSO or 2.5 or 5 μM PT for 72 hr. Cellular lysates were prepared and analyzed by RPPA. The results, presented as a heat map with a list (Fig 3A), indicated significant differences in protein expression.

To identify the signaling networks modulated by PT, proteins showing significant differences (P<0.05) were further analyzed using a web-based bioinformatic program. KEGG (Kyoto Encyclopedia of Genes and Genomes) pathway analyses (www.genome.jp/kegg) revealed that the ATM/ATR-CHK1/2-p53 pathway is activated by PT in A549 cells (S1 Fig).

These results are consistent with our observation that PT treatment results in proliferation inhibition and S-phase arrest (Fig 2), indicating that low PT concentrations induce replicative stress that results in activation of the ATR/ATM-CHK1-p53 axis. We further confirmed CHK1 and p53 activation via Western blot analysis. As shown in Fig 3B, treatment with low PT concentrations induced CHK1 and p53 phosphorylation, which significantly enhanced the proliferation of cells cultured in serum-free medium. Interestingly, however, no change in the level of p21, which is a target of p53 and implicated in G1 arrest, was noted. This result is in contrast with other studies demonstrating that treatment of human cells with PT activates p53 and increases p21 expression, resulting in G1 cell cycle arrest at higher concentrations. Taken together, a low concentration of PT leads to replicative stress, likely inhibiting the replication machinery and resulting in S-phase arrest.[43].

### p53-dependent cell growth inhibition effects of PT in precancerous human bronchial epithelial cell lines

**Fig 4:**
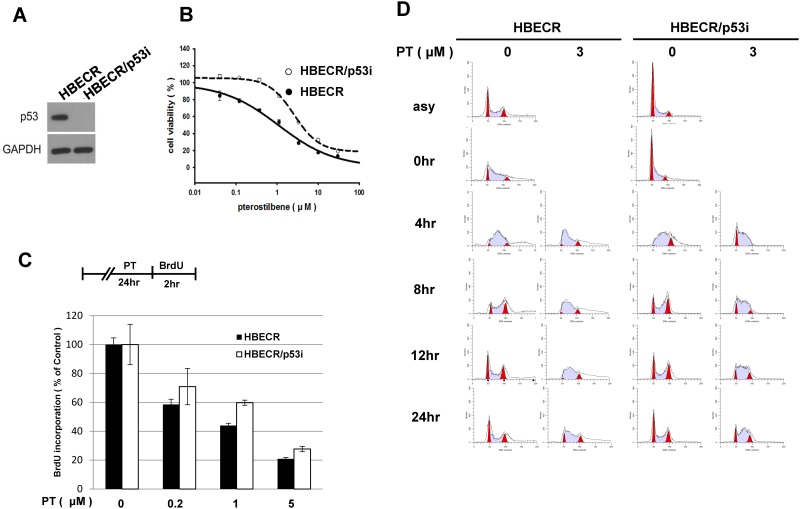
Enhanced cytotoxic effects of pterostilbene in precancerous cell lines, HBECR and HBECR/p53i. (A) Western blotting analysis showing the presence and absence of p53 in HBECR and HBECR/p53i cell lines. (B) Cell viability of HBECR and HBECR/p53i cells (2×10^3^) treated with different concentrations of pterostilbene ranging from 0 to 100 μM. (C) Cell proliferation ELISA assay with BrdU incorporation. (D) HBECR and HBECR/p53i cell lines were synchronized by double thymidine block. The HBECR cells were only partly synchronized. Cells were released into S phase along with treatment of pterostilbene at the final concentration of 3 μM. At the indicated time point from the thymidine block, cells were harvested and fixed for cell cycle analysis.

Based on proteomic analyses of A549 cells grown in the absence of serum, treatment with a relatively low PT dose induces p53 activation, leading to growth inhibition and S-phase arrest. To further investigate the chemopreventive effects of PT and to determine whether the ATM/ATR/CHK/p53 signaling pathway plays an important role in the cytotoxic effects of PT, we performed cell survival assays with molecularly defined precancerous human bronchial epithelial cells, HBECR and HBECR/p53i cell lines, with a low dose of PT. The precancerous human bronchial cell line HBECR was previously established by expressing cdk4, a catalytic subunit of telomerase (hTERT), and the oncoprotein KRAS^V12D^ in normal primary human bronchial cells.[44] Although these cell lines underwent a few oncogenic changes, they are not completely transformed because they fail to form tumors in immunodeficient mice. The only difference between these two lines, HBECR and HBEC/p53i is that HBECR cells express a functional p53 but HBECR/p53i cells do not (Fig 4A). [44]

Both HBECR cell lines are sensitive to low doses of PT determined by whole cell count and by ELISA with BrdU incorporation. Of interest, HBECR cells are more sensitive than HBECR/p53i cells to low doses of PT (Fig 4B and 4C), suggesting that the cytotoxicity of low-dose PT is maximized in the presence of a functional p53 protein. To understand the effects of low PT doses on cell cycle progression, HBECR and HBECR/p53i cells were synchronized via double thymidine block, and FACS analyses were performed at the indicated times after release. Thymidine blocking in these premalignant cells was not as efficient as in cancer cells (Fig 4D, asynchronized vs. 0 hr). However, majority of released HBECR cell population was arrested in early S phase upon PT treatment up to 24 hrs. (Fig 4D, 4hr, 8hr, 12hr) To a lesser extent, the PT treated HBECR/p53i cell cycle was also arrested in S phase for hours. At 24hr, most of PT treated HBECR cells failed to return to regular cell cycle as the control treated cells while some PT treated HBECR/p53i cells catch up the normal cell cycle. (Fig 4D, 24hr)

Taken together, these findings suggest that replication stress induced by low doses of PT activate p53 in S phase and that this activation leads to delayed cell cycle progression. Therefore, PT could serve as a potential chemopreventive agent that prevents the progression of precancerous cells to cancerous cells in a p53-dependent manner.

### Treatment with low concentrations of PT leads to activation of the ATM/ATR-CHK1-p53 pathway, causing S-phase arrest in precancerous cell lines

**Fig 5:**
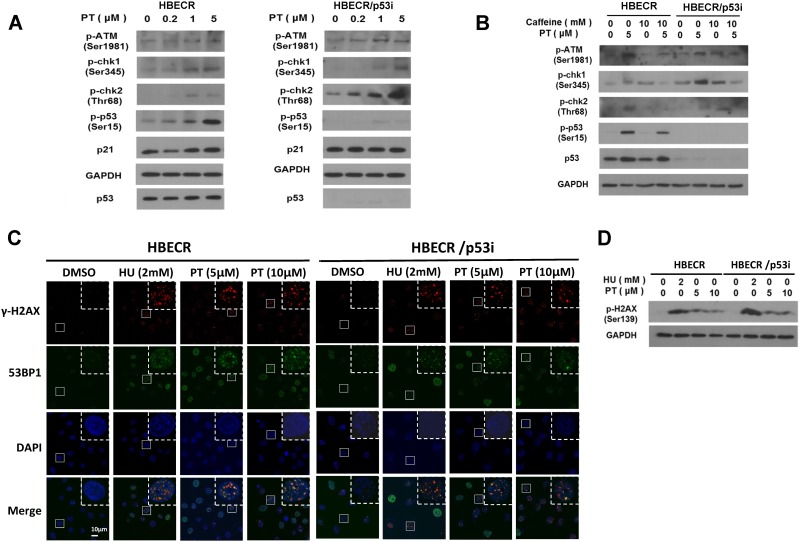
Activation of ATR-chk1/2-p53 pathway induced by pterostilbene in pre-malignant lung epithelial cell lines. (A) Cultured HBECR and HBECR/p53i cells were treated with different concentrations of pterostilbene ranging from 0 to 5 μM. After 24 hours, cell lysates were prepared and the proteins were visualized by Western blotting using specific antibodies as indicated. (B) HBECR and HBECR/p53i cells were treated with indicated concentration of ATM inhibitor caffeine and/or pterostilbene for 24 hr. (C) HBECR and HBECR/p53i were treated with ATM activator hydroxyurea (HU) and/or pterostilbene (PT) for 24hr. γH2AX and 53BP1 were detected by immunofluorescence analyses. The images were taken at 63X with confocal microscope. The wide view of the images are in the supplementary figure (S2 Fig). (D) Phosphorylation of H2AX upon HU (hydroxyurea) or PT was determined by Western blot analysis.

We further examined the mechanism underlying p53 phosphorylation upon PT treatment and observed that the phenomenon in A549 lung cancer cells was also observable in precancerous cell lines. To determine whether p53 phosphorylation is caused by replicative stress in HBECR and HBECR/p53i cells upon treatment with low doses of PT, activation of proteins involved in the checkpoint was analyzed by Western blotting at 24hrs or PT treatment. As shown in Fig 5A, the ATM/ATR-CHK1/2 axis is activated upon treatment with low doses of PT, an effect that is indicative of replication stress.

**Fig 6:**
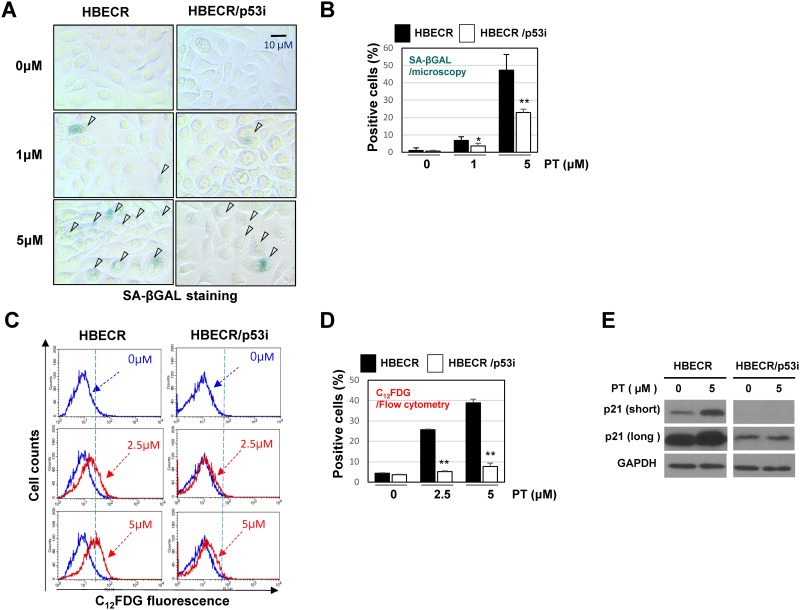
p53 dependent senescence by pterostilbene in precancerous bronchial cell. (A) Induced senescence on HBECR and HBECR/p53i cells by pterostilbene. The cells on glass cover slips were treated with pterostilbene (PT) for 72hr and then stained in situ for senescence-associated β–galactosidase. The representative images are shown (top left). The cells cultured were also treated with PT for 72hr and then collected for C_12_FDG staining followed by analysis with flow cytometry. (bottom left). The SA-βgal positive cells were quantified by microscopically or flow cytometry. *, *P < 0*.05; **, *P< 0*.*01* by Student’s *t* test. (B) Change of p21 and p53 by 72hr treatment of PT (5μM).

However, p21 levels were not significantly altered at 24hr, indicating the phosphorylated p53 did not results in transcriptional activation of p21 yet (24hrs after PT treatment).Given that HBECR/p53i cells are depleted of p53, p53 was minimally detectable by Western blotting. In contrast, increased CHK1 and CHK2 phosphorylation was observed in HBEC/p53i cells. Although the molecular mechanism for enhanced CHK2 activation is currently unclear, this effect might due to other p53-independent checkpoints being overridden.[45] To examine whether phosphorylation of CHK1, CHK2 and p53 in response to PT is dependent on ATM/ATR, HBECR and HBECR/p53i cells [46] were treated for 24 hrs with both PT and caffeine, an ATM/ATR inhibitor. As shown in Fig 6B, caffeine treatment reversed the PT-induced activation of CHK1, CHK2 and p53, demonstrating that the chemopreventive effects of PT are mediated by ATM/ATR. Given that ATM activity can be represented by γH2AX, we also examined expression of γH2AX by cell staining and found it to be increased focal stained regions upon treatment with PT (Fig 5C). The PT induced γH2AX nuclear foci were well co-localized with 53BP1, known to make repair complex, which is widely accepted to localize at sites of DNA strand breaks.[47] Hydroxyurea was also used as positive control to generate replication stress in S phase.

Because treatment of both HBEC and HBEC/p53i cells with low doses of PT induced S-phase arrest and HBECR cells exhibited increased sensitivity compared with HBECR/p53i cells, we determined whether the enhanced cytotoxicity is due to the presence of a functional p53 protein, thereby leading to increased apoptosis or senescence in the HBECR cell line. To this end, we used flow cytometry to compare the levels of apoptosis between HBECR and HBECR/p53i cells after treatment with low doses of PT. Of interest, low-dose PT did not induce apoptosis in HBECR or HBECR/p53i cells, a result that was confirmed in A549 cells (S3 Fig).

Therefore, the cytotoxic effects of low doses of PT are potentially mediated via p53-dependent inhibition of DNA replication at S phase but not by apoptosis induction. Nonetheless, cell senescence was well induced by 72 hrs treated PT in HBECR cells than in HBECR/p53i cells, in a dose dependent manner in two different assays. (Fig 6A, 6B, 6C and 6D) A senescence inducer, p21 and p53 amount were increased in HBECR but while did not change in HBECR/p53i cells. (Fig 6E) These results strongly suggest that the p53 dependent cytotoxic effect by PT is mainly through replication stress activated p53-p21 mediated senescence.

**Fig 7:**
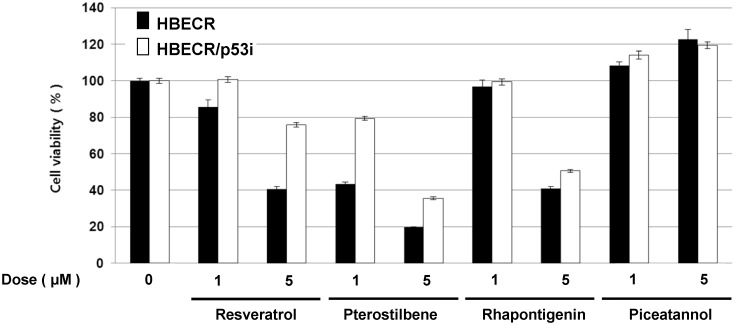
p53 dependent effects of pterostilbene in precancerous bronchial cell senescence. The p53 dependent cytotoxic effects of resveratrol, pterostilbene, rhapontigenin and piceatannol on precancerous bronchio-epithelial cells, with or without functional p53. *, *P < 0*.05; **, *P< 0*.*01* by Student’s *t* test.

Among several stilbenoids, PT exhibits the greatest cytotoxicity in NSCLC cells (shown in Fig 1). Thus, we assessed whether p53-dependent cytotoxicity in these precancerous cells is a unique feature of PT by performing survival assays with relatively low doses of the stilbenoid analogues PT, resveratrol, rhapontigenin, and piceatannol. PT exhibited the most profound cytotoxic effects in a p53-dependent manner (Fig 7).

## Discussion

Here, we provide evidence that pterostilbene is a potential chemopreventive phytochemical working with a functional p53. The low dose of PT induced replicative stress, resulting in cell cycle arrest at S phase and activation of the ATM-CHK-p53 axis, followed by senescence. To the best of our knowledge, this is the first report to clarify the replication stress signaling-dependent chemopreventive function of pterostilbene in precancerous bronchial cell lines. Our findings are distinguished from previous findings by the following. First, cytotoxic effects can be achieved with low concentrations of PT. This result is in sharp contrast to that observed with a high dose of PT (usually greater than 50 μM), which causes G1 cell cycle arrest and overexpression of p21, with subsequent apoptosis via the mitochondrial and Fas/FasL pathways.[48] Second, the cytotoxic effects of PT are dependent on functional p53 during S phase. Enhanced p53 phosphorylation was noted with low doses of PT. The accumulation of p53 and p21 was evident at 72hr PT treated HBECR and associated massive senescence in only p53 intact cells. Of interest, increased CHK2 phosphorylation was noted in the absence of p53 in HBECR/p53i cells, indicating that replication stress is induced by low doses of PT in the absence of p53.

Enhanced proliferative pressure and genomic instability of both precancerous lesions and cancers generates a considerable amount of spontaneous DNA damage. In precancerous cells, this accumulated DNA damage induces cell cycle arrest, senescence and apoptosis via p53 activation. However, such barriers bypassed in cancer cells by various mechanisms, including p53 mutations that subsequently impair DNA damage responses.[49–51] Therefore, precancerous cells could serve as a potential target of cancer prevention by inducing catastrophic genomic instability followed by cell death or cell cycle escape. [52, 53]

The underlying mechanism by which PT induces G1-arrest and apoptosis at high doses (according to the reports of others) and S-phase arrest at low doses (in the current research) remains unclear. RPPA, Western blot analysis and cyto-immunostaining demonstrated that low-dose PT activates ATM kinase activity followed by the CHK-p53 pathway, potentially as a result of replicative stress. The co-localization of 53BP1 and γH2Ax foci upon PT treatment demonstrated further that problematic replication are generated by PT in S phase cells. However, such events raise an important question: what induces this replicative stress? One possibility is that PT inhibits the function of the DNA replication machinery during S phase, resulting in collapse of the replisome.[54] Another possibility is that PT indeed functions as a genotoxic agent that is also capable of inhibiting DNA replication and causing DNA replication forks to collapse. We speculate that the latter might be the case, as it was recently reported that resveratrol, an analogue of PT, induces DNA double-strand breaks by inhibiting the function of TOPO II.[25]. It might be possible PT sensitized DNA damage responding machinery (ATM/ATR network) detecting spontaneous replication stresses. In contrast, high doses of PT induce cell cycle arrest at G1 phase when the DNA replication machinery is not activated and led apoptosis.[55] Our data showed that PT gives replication stress to the premalignant cells at the S-Phase. The mild replication stress induced chromosomal lesions are transmitted to daughter cells [56] without fixing the problem. Accordingly, PT induced massive chromosomal lesions in G1 phase should be responsible for the p53 induced senescence.

Our findings that low-dose PT exhibits chemopreventive activity are noteworthy. PT is a dietary phytochemical, and thus the best method of PT uptake involves consuming fruits and vegetables that contain PT, including blueberries. However, although high doses of PT cannot be obtained from foods because these dietary sources contain a trivial amount of PT, most functional studies of PT have been performed with high concentrations. Therefore, the biological effects of PT that we observed at lower doses may be more meaningful. The induction of replication stress in the precancerous cells by diet may be though to be harmful. However, recent findings that human cells activate an ATR/ATM-regulated DNA damage response network to prevents carcinogenesis [57, 58] support the idea that the activation of this signal may contribute to the chemoprevention.

Another finding that PT showed a better effect when the cancer cell line was not supplemented with FBS, which contains many undefined growth factors, is also noteworthy. Most cells in solid tumors are in a microenvironment that involves a limited blood supply due to the distance from blood vessels. Therefore, these cells may depend on paracrine and autocrine growth factors more. In the same regard, cells cultured under serum-free conditions with the limited supplementation may represent in vivo tumor cells. The superior cytotoxic and senescence effects of PT compared to other stilbenoids also may be important. p53 mutation is one of the most important genetic changes in lung squamous carcinogenesis, and the relatively superior effect of PT on p53-intact precancerous cells compared to on p53-suppressed cells also suggests the potential of PT to serve as a chemopreventive agent for squamous lung carcinogenesis at the early stage, before p53 mutation occurs.

Here, we demonstrated using an unbiased proteomic assay and molecularly defined lung carcinogenesis model that a stilbenoid, PT, exerts unique p53-dependent chemotherapeutic effects through the ATM/CHK/p53 tumor suppressive pathway leading to cell senescence. To the best of our knowledge, this is the first study to demonstrate PT-specific p53-dependent chemopreventive activity at the molecular level. Based on our results, we propose that absorption of PT from dietary sources in an effort to suppress lung cancer development should to be initiated as early as possible because the effect may be decreased when p53 is already inactivated.[59]

## Supporting Information

S1 Fig Pathway diagrams visualized by KEGG by Kyoto Encyclopedia of Genes and Genomes program. Changes in protein expression levels acquired from A were applied to bioinformatics database to determine activated or downregulated pathways upon pterostilbene treatment.
(TIF)

S2 Fig Enlarged figures from the Fig 5C. Immunofluorescent analysis of gH2AX and 53BP1 foci formation upon HU and PT treatment. gH2AX and 53BP1 form distinctive foci and were co-localized together. Confocal microscope (Leika) used with 63X objectives.
(TIF)

S3 Fig Low concentration of pterostilbene does not induce apoptosis FACS analysis of A549 (A) and HBECRs (B). Cells were treated with indicated concentration of pterostilbene for 72 hours. Cells were harvested, fixed and stained with annexin V-FITC and propidium iodide for FACS analysis. Numbers in the boxes indicate the percentage of annexin V positive cells.
(TIF)
