# Supplementary figures and images for: ATM/CHK/p53 Pathway Dependent Chemopreventive and Therapeutic Activity on Lung Cancer by Pterostilbene
Source: PLoS One. 2016 Sep 9;11(9):e0162335. doi: 10.1371/journal.pone.0162335 (PMC5017581; doi:10.1371/journal.pone.0162335)

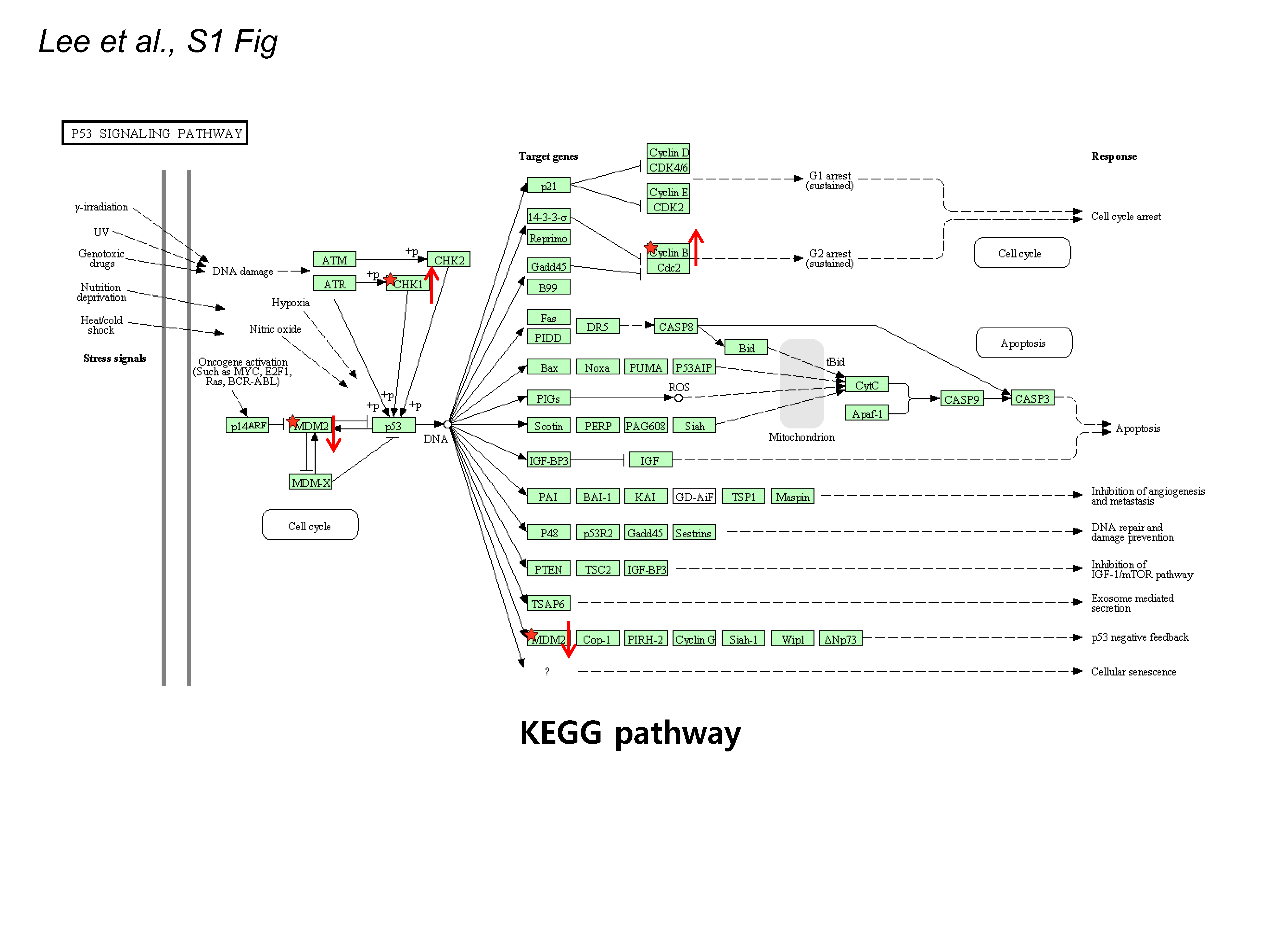

Supplement: S1 Fig — Changes in protein expression levels acquired from A were applied to bioinformatics database to determine activated or downregulated pathways upon pterostilbene treatment. (TIF) [file pone.0162335.s001.tif]

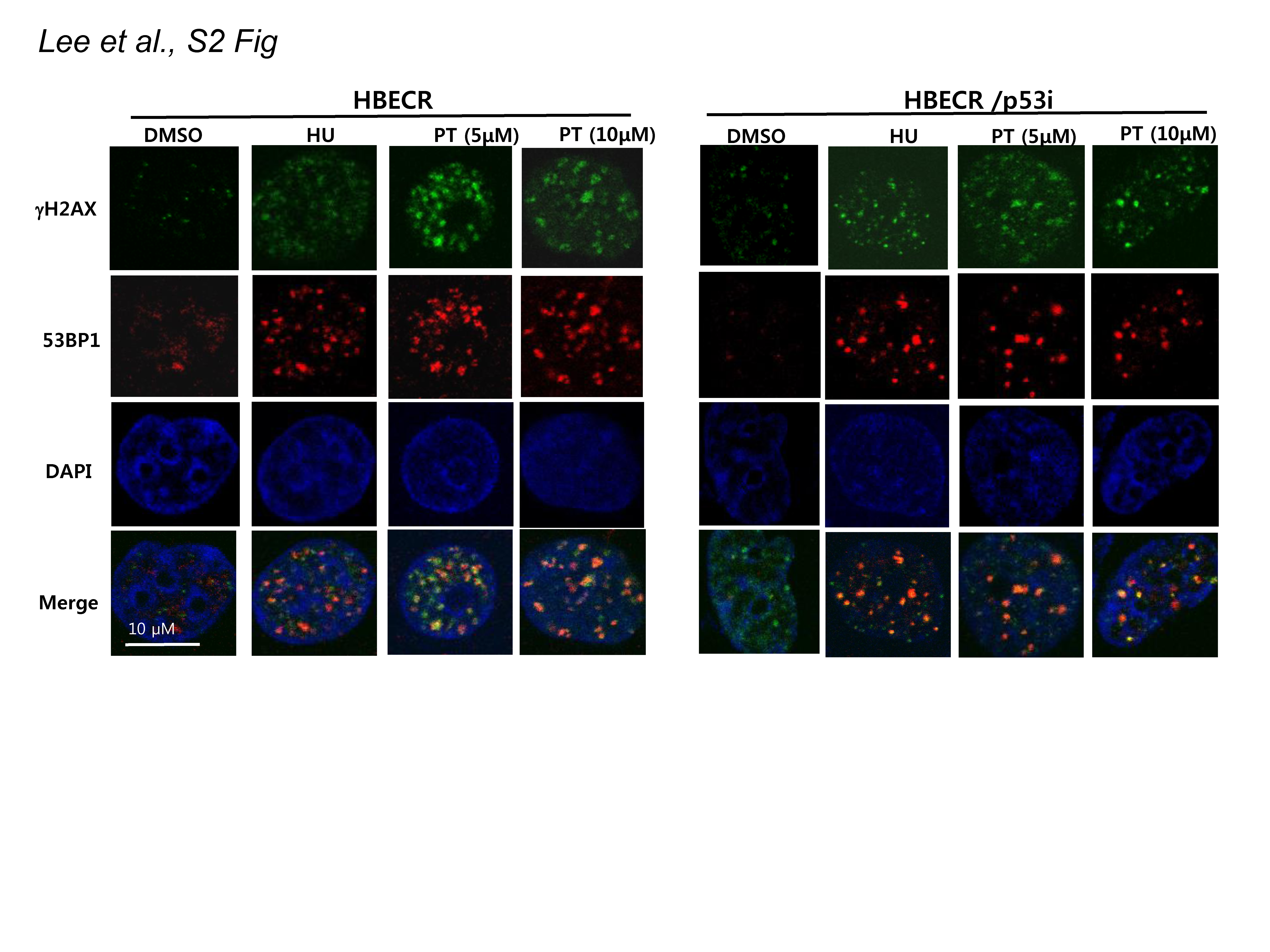

Supplement: S2 Fig — Immunofluorescent analysis of gH2AX and 53BP1 foci formation upon HU and PT treatment. gH2AX and 53BP1 form distinctive foci and were co-localized together. Confocal microscope (Leika) used with 63X objectives. (TIF) [file pone.0162335.s002.tif]

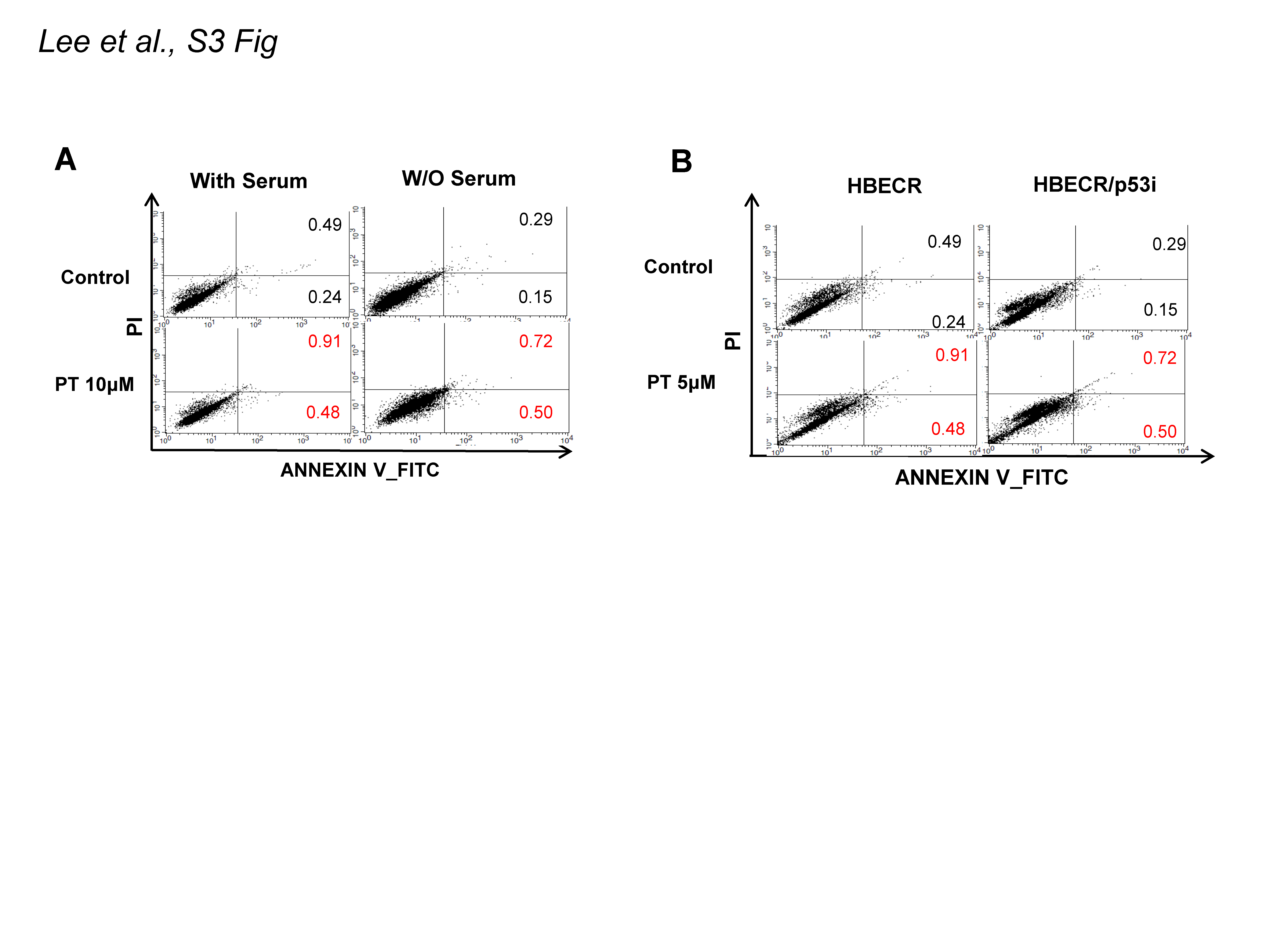

Supplement: S3 Fig — Cells were treated with indicated concentration of pterostilbene for 72 hours. Cells were harvested, fixed and stained with annexin V-FITC and propidium iodide for FACS analysis. Numbers in the boxes indicate the percentage of annexin V positive cells. (TIF) [file pone.0162335.s003.tif]
